# A Case of an Ulnar Nerve Laceration With Distal Humerus Fracture From Machete Trauma

**DOI:** 10.7759/cureus.28737

**Published:** 2022-09-03

**Authors:** Max Murray-Ramcharan, Jared Atchison, Ofelia Leroux, Ryan Engdahl

**Affiliations:** 1 General Surgery, Harlem Hospital Center, Harlem, USA; 2 Surgery, Columbia University, New York, USA; 3 Plastic Surgery, Harlem Hospital Center, Harlem, USA

**Keywords:** complex fracture, peripheral nerve transfers, distal humerus fracture, upper extremity trauma, peripheral nerve surgeries

## Abstract

Ulnar nerve dysfunction following distal humerus fractures is a recognized phenomenon. There is no dominating consensus regarding the optimal management of the ulnar nerve during surgical intervention for these fractures between leaving the nerve in situ versus nerve transposition for better healing. Additional complexities arise in the case we present, in which there was an open fracture compounded with an ulnar nerve laceration from a traumatic injury with a machete knife. We review and discuss the management of ulnar nerve injuries associated with complex open fractures of the humerus for optimizing patient outcomes following these injuries.

## Introduction

Machete trauma of the extremities can inflict significant injury. Lacerations extend deeply into soft tissues and other structures, which can create additional complexities in management. Although these injuries may not be common, reconstructive surgeons may be required for deep complex injuries from a machete [1,2]. In addition to injury management, a review of 48 patients documented social factors including psychiatric history and substance and tobacco use in those assaulted using a machete, which may create further challenges in managing these patients [1]. We review a case of a homeless individual with upper extremity machete trauma. Injuries at the elbow included lacerations of the ulnar nerve, soft tissues, and triceps muscle, as well as a humeral fracture. We discuss the case and highlight complexities of such injuries and highlight management of the ulnar nerve.

## Case presentation

**Figure 1:**
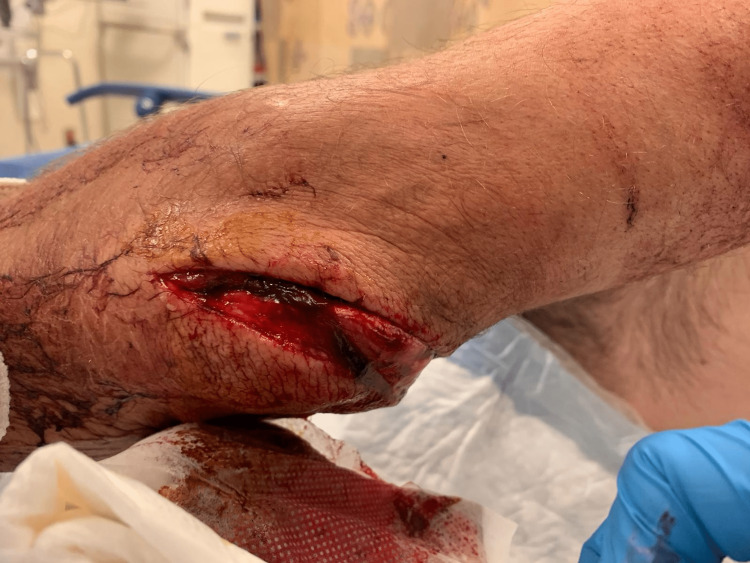
Initial patient presentation showing laceration incurred by a machete on the left elbow

A 40-year-old homeless male patient with polysubstance use was admitted to the hospital after being attacked with a machete and thus sustaining a deep complex laceration of the elbow (Figure 1).

**Figure 2:**
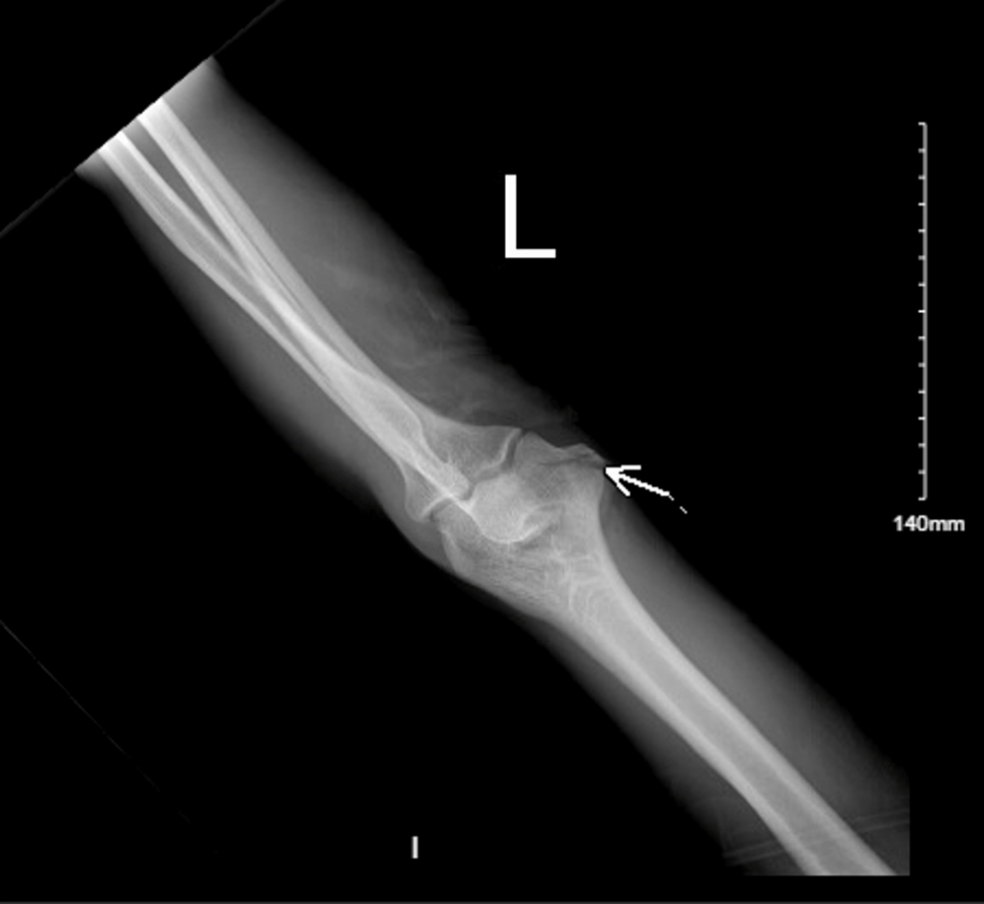
Initial X-ray of the left elbow with medial epicondyle fracture (white arrow)

On physical examination, in addition to a 10cm long, 3cm deep laceration on the posteromedial aspect of the left elbow, he was noted to have an inability to extend the forearm as well as impaired finger motion of the fourth and fifth fingers with loss of abduction and adduction and lack of sensation on the ulnar side of the hand and fingers. He had strong pulses in both radial and ulnar arteries and good capillary refill throughout. Radiographs showed a lucency of the left medial epicondyle compatible with a non-displaced fracture (Figure 2).

**Figure 3:**
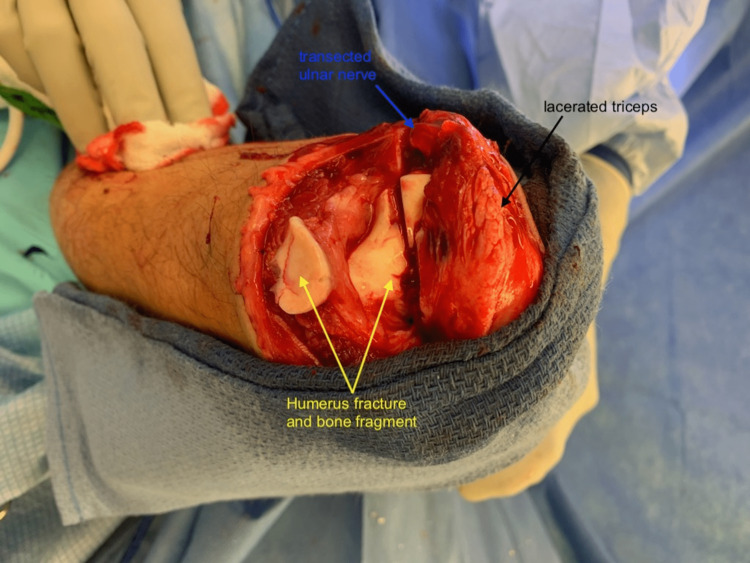
Intraoperative findings of triceps tendon laceration, left distal humerus fracture, and ulnar nerve transection Injuries to the left triceps, left ulnar nerve, and left humerus labelled

He underwent immediate operative exploration, which revealed a traumatic arthrotomy at the left elbow with triceps tendon laceration as well as an intra-articular left distal humerus fracture and complete ulnar nerve transection without significant defect (Figure 3).

**Figure 4:**
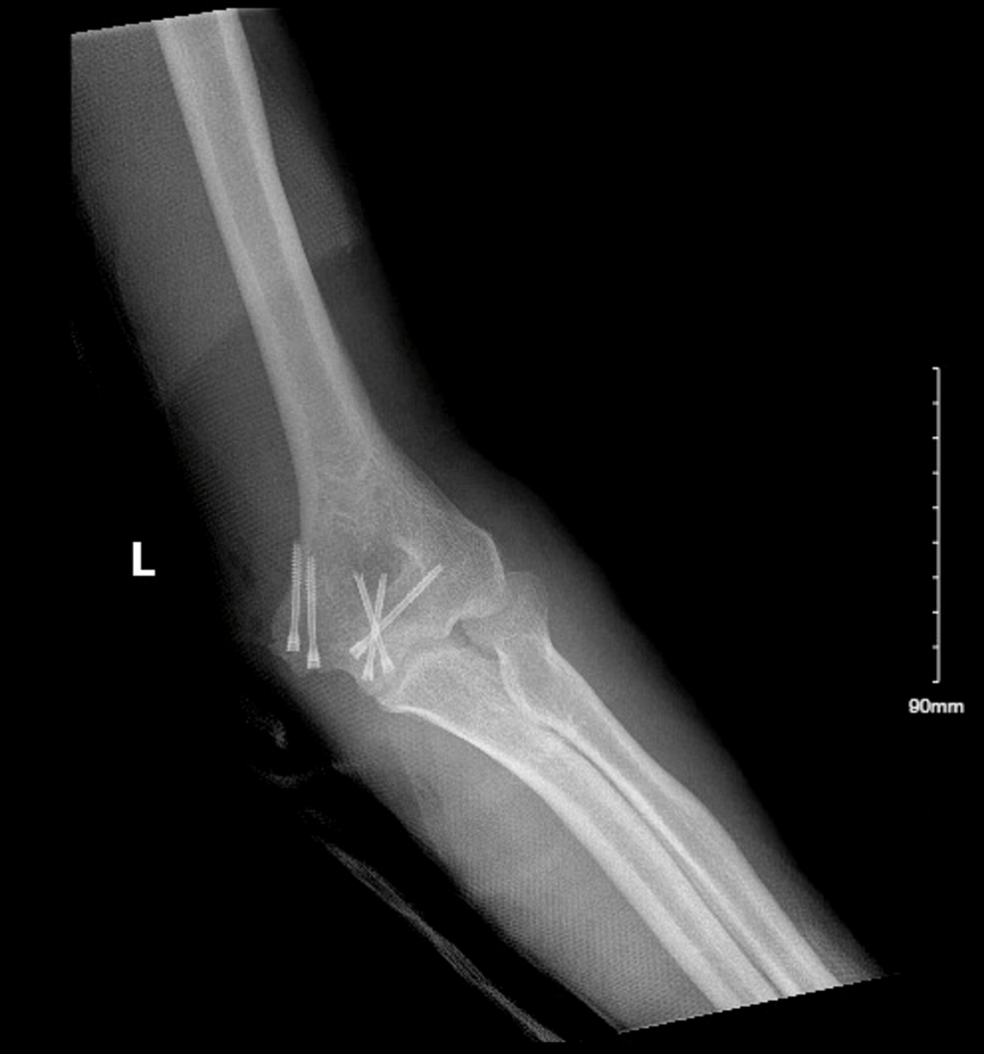
X-ray showing postoperative imaging of left humerus fracture repair

Procedures done included thorough irrigation of wounds and debridement of the left elbow traumatic arthrotomy. Open reduction and internal fixation of displaced intra-articular distal humerus fracture was performed using headless cannulated screws, three in the median epicondyle and two across the trochlea with restoration of intra-articular anatomy (Figure 4).

**Figure 5:**
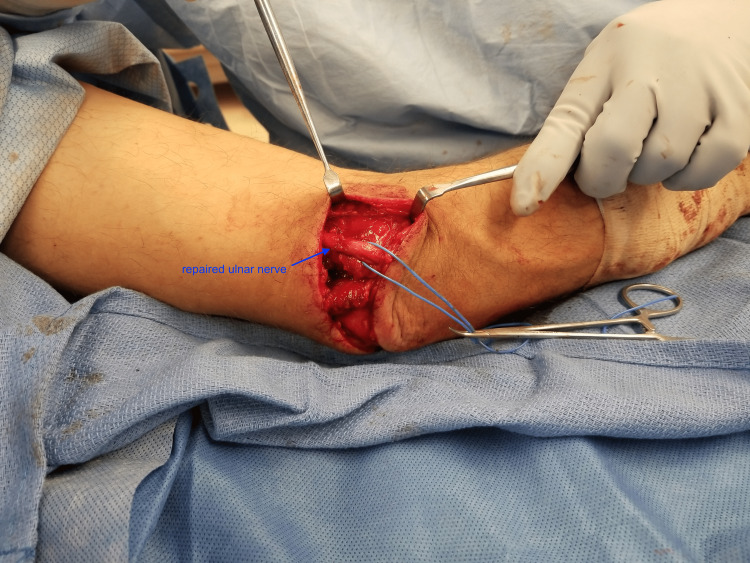
Repair of the ulnar nerve with transposition Vessel loop around the repaired left ulnar nerve

Repair of triceps tendon laceration was then completed with suture anchor and fiberwire, and attention was turned to the transected ulnar nerve. The proximal ulnar nerve was meticulously dissected from within Guyon's canal and the distal segment noted just distal to Osborne's fascia. We elected to proceed with an ulnar nerve repair and transposition due to the extensive soft tissue injuries and fractures at the surgical site in order to take any excess tension off of the repair, as well as to limit any scarring to allow for ease of subsequent future nerve transfer procedures. The ulnar nerve was repaired primarily with 10 circumferential 8-0 epineural nylon sutures using surgical microscope without tension noted (Figure 5).

A subcutaneous fascial sling was then developed just beyond the region of injury, and the nerve transposed and secured with 3-0 Biosyn™ sutures. There was no significant tension noted after transposition, and there was good freedom of the nerve with movement of the arm and elbow joint, with no subluxation of the ulnar nerve with the elbow flexion test. The wound was irrigated once again, and after closure of the laceration, the arm was splinted with an elbow splint with flexion to 90 degrees. The patient was evaluated postoperatively and noted to have mildly improved physical examination findings than before, with decreased sensation and motor function of the left fourth and fifth digits. Unfortunately, the day following the repair, the patient left against medical advice and was unable to be located for follow-up.

## Discussion

Extremity trauma from machete can inflict significant injury with multiple combined soft tissue and bone injuries, which may impart complexities in management. Prominent social factors including being undomiciled, substance use, and documented psychiatric history may further challenge management in those suffering from assault and trauma. An interesting part of the operative case involves the management of ulnar nerve injury. Injuries such as distal humerus fractures around the elbow may involve in situ repair or, at times, transposition of the nerve. In general, optimal handling of the ulnar nerve, whether to release and leave in place or transpose the nerve, at the time of repair of distal humerus fractures remains controversial [3]. Although routine transposition might increase the chance of morbidity such as nerve dysfunction [3-6] and also have an increased likelihood of ulnar neuritis as compared to alternatives [4], differing mechanisms and other traumatic factors are needed to be considered in select cases for optimal nerve handling. Although there is a lack of consensus on the definitions of ulnar nerve instability, factors typically influencing their decision to proceed with anterior transposition of the ulnar nerve are subluxation on examination. In cases of nerve compression such as in cubital tunnel with muscle atrophy, nerve transposition is currently considered. As there are data lacking for ulnar nerve laceration with soft tissue injury and transposing the nerve in a complex laceration such as in our case, we highlight this case and discuss management of the nerve in this setting. We chose to transpose the nerve to aid in nerve healing, without tension, as well as to place the nerve repair in an area outside the complex soft tissue and triceps repairs and humerus fracture. This would likely produce a less localized inflammatory response and decreased scar tissue formation, assisting in future planning for a possible nerve transfer including a supercharged end-to-side anterior interosseous to ulnar motor nerve transfer for intrinsic musculature re-innervation [7]. This planned procedure would be beneficial in this case of a high ulnar nerve injury to reduce long-term dysfunction of forearm and hand muscles and cubital tunnel syndrome. Additional benefit of transposition may be attributed to increased nerve overlap during repair to assist in healing and regain of function. [8]. Unfortunately, the patient was lost to follow-up for us to perform this and track the repairs.

## Conclusions

Machete extremity trauma can inflict significant injury to multiple structures and require complex multidisciplinary care for treatment and repair. In our case, there were soft tissue injuries including triceps tendon lacerations, lacerated ulnar nerve, and a humerus fracture. Regarding ulnar nerve repair, although no studies have specifically reviewed ulnar nerve transposition following laceration repair with concomitant humeral fractures, we chose to transpose the nerve in our case to aid in nerve healing without tension as well as to place the nerve in an area outside the complex humeral and triceps repairs. Further studies examining optimal repair of essential nerves in the setting of complex injury is warranted. Additionally, it is worth exploring on a larger scale the social risk factors for leaving against medical advice and complex upper extremity lacerations.
